# Tropical dry forest land use/land cover change detection using semi-supervised deep learning algorithms and remote sensing

**DOI:** 10.1007/s10661-025-14897-4

**Published:** 2026-02-02

**Authors:** Juan C. González-Vélez, Maria C. Torres-Madronero, Juan D. Martínez-Vargas, Paula Rodríguez-Marín, Jheison Perez-Guerra, Veronica Herrera-Ruiz

**Affiliations:** 1 Instituto Tecnologico Metropolitano (ITM), Calle 54a No. 30-01, Medellin, 050012 Colombia; 2 Department of Computer and Decision Sciences, Universidad Nacional de Colombia, Av. 80 No. 65-223, Medellín, 050034 Colombia; 3 Universidad EAFIT, Carrera 49 No. 7 Sur-50, Medellín, 050022 Colombia

**Keywords:** Land use land cover, Multispectral image, Synthetic aperture radar, Semi-supervised learning, Deep learning, Tropical dry forest

## Abstract

Tropical dry forests (TDFs) provide essential ecosystem services yet are notoriously difficult to map using remote sensing data due to their spectral similarity to open fields, variability in forest regrowth, and factors such as seasonal leaf phenology and landscape fragmentation that hinder their discrimination. These limitations are critical in data-scarce regions where traditional classification methods struggle. This study introduces a novel semi-supervised deep learning (DL) framework for land use and land cover (LULC) change detection, combining synthetic aperture radar (SAR) and optical satellite imagery for TDF change detection. The proposed framework combines unsupervised pseudo‑labeling and a custom Y‑Net architecture to fuse optical and radar imagery, enabling accurate change detection with limited labeled data. The framework achieves state-of-the-art results, with a mean overall accuracy of 95.3% and a mean Intersection over Union (mIoU) of 88.1%, outperforming established models like standard U-Net and PSPNet. Even in scenarios where only 60% of the dataset is labeled, the semi-supervised method maintains accuracy above 90%, demonstrating its robustness in limited-data conditions. The proposed semi-supervised framework is applied to reveal TDF changes in the Cauca River Valley in Antioquia (Colombia) using satellite images between 2017 and 2021. These findings provide a valuable foundation for advancing remote sensing applications in environmental monitoring, conservation planning, and sustainable resource management.

## Introduction

Land use and land cover (LULC) changes are among the most important representations of the relationship between humans and the environment. It is considered a dynamic component of landscape analysis and one of the main drivers of global environmental change. Identifying and monitoring LULC change is vital for adequate land and resource management (Gaur & Singh, 2023).

One of the world’s most diverse and threatened biomes is the tropical dry forest (TDF) (Murphy & Lugo, 1986). TDF features a pronounced seasonality, resulting in high heterogeneity of vegetation types and adaptation strategies (Murphy & Lugo, 1986), and provides vital ecosystem services like carbon sequestration, water regulation, soil conservation, and biodiversity maintenance (Aide et al., 2012). However, TDFs are also highly vulnerable to human activities, such as agricultural expansion, logging, mining, and urbanization, leading to severe fragmentation and degradation (Miles et al., 2006). Thus, monitoring TDF changes is essential (Rivas & Navarro-Cerrillo, 2024).

Remote sensing is a powerful tool for acquiring timely and spatially consistent information about the Earth’s surface (Sánchez-Cuervo et al., 2012). Although optical imagery is widely used for LULC classification due to its high spectral resolution, it faces limitations such as the dependence on solar illumination, susceptibility to atmospheric effects, and low sensitivity to vegetation’s structural and phenological variations (Li et al., 2023; Xie et al., 2008). Synthetic aperture radar (SAR) overcomes these limitations by utilizing active microwave radiation to penetrate clouds and haze, operating day and night, and capturing geometric and dielectric properties of the targets (Kechagias-Stamatis & Aouf, 2021).

Despite extensive research on LULC change detection using remote sensing data, existing methods for TDF mapping rely mainly on single-modality classification or classical machine learning models, yielding only moderate accuracy and limited generalization (Castro et al., 2003; Hernández-Stefanoni et al., 2020). The spectral responses of TDFs often overlap with open fields, and secondary forest stands exhibit wide variability, making classification particularly challenging (Helmer, 2000). Severe fragmentation and degradation of TDF patches produce heterogeneous LC, further hindering accurate mapping (Rivas & Navarro-Cerrillo, 2024). Recent works have started to explore SAR-optical fusion and deep learning (DL) to address these issues (Shimizu et al., 2019), but an integrated framework tailored to TDFs is still missing.

Obtaining cloud-free optical images is especially challenging for accurately identifying TDF in countries like Colombia, where the seasonal migration of the Intertropical Convergence Zone (ITCZ) and humid maritime currents produce near-continuous cloud cover. During the wet season, persistent cloudiness limits the availability of high-quality satellite data when TDF vegetation is leaf-on (Castillo et al., 2012). Conversely, clear skies may coincide with a leafless canopy in the dry season, reducing vegetation signals and complicating TDF discrimination (De la Barreda-Bautista et al., 2011).

Besides challenges from cloud cover and limited optical imagery, the low reflectance of dry-season TDF vegetation hinders accurate classification. Integrating multi-season optical data, for example, combining leaf-on wet-season scenes with leaf-off dry-season scenes which leverage phenological variability to enhance land-cover separability, but this strategy remains underused in TDF mapping. While radar-optical image fusion has been suggested to mitigate classification issues (Clerici et al., 2017; Herrera-Ruiz et al.,  2023; Mullissa et al., 2023), yet most prior studies treat each season separately and do not fully exploit cross-seasonal fusion. Moreover, DL approaches that could significantly improve classification accuracy, particularly in semi-supervised settings (Mohsenifar et al., 2021), remain underexplored for TDFs.

DL semantic segmentation has recently been applied to LULC mapping, demonstrating improved accuracy in comparable settings (Yuan et al., 2023). However, classification often hinges on substantial annotated training data, which is scarce in remote sensing. Nonetheless, few studies have leveraged such methods for TDF, and research applying semi-supervised learning (SSL) remains scarce. Recent SSL frameworks, such as (Chen et al., 2023; Hafner et al., 2023), emphasize both the potential of SSL and its limited adoption.

Several key challenges hinder the accurate monitoring of TDFs, forming the primary research gaps this study addresses. First, obtaining cloud-free optical images in tropical regions like Colombia is exceptionally difficult due to persistent cloud cover, limiting the availability of high-quality data. Second, TDFs are spectrally ambiguous and often overlap with other LULC classes, making them difficult to discriminate. Third, while deep learning (DL) and SAR-optical fusion show promise, an integrated framework specifically tailored to TDFs is still missing. Finally, the most advanced DL models require substantial annotated training data, which is scarce in remote sensing, yet the use of semi-supervised learning (SSL) to overcome this data scarcity remains underexplored for TDF mapping.

This study aims to overcome these limitations. The main contributions and novelties of this work are explicitly listed as follows:

- A novel dual-encoder Y-Net architecture adapted from Lan et al. (2020) for remote sensing. This architecture is specifically designed to fuse multi-resolution optical (5 m) and SAR (10 m) data natively, without requiring resampling, by incorporating an early-fusion module to exploit complementary spectral and structural information.
- An integrated semi-supervised (SSL) framework that combines the Y-Net architecture with unsupervised pseudo-labeling. This approach addresses the critical challenge of labeled data scarcity, common in remote sensing applications.
- The first application and validation of such a semi-supervised, multi-modal (SAR-Optical) deep learning framework for TDF LULC change detection in the challenging, cloud-prone Cauca River Valley of Colombia.
- This work’s hypothesis is that this combination of optical-radar fusion and SSL training will improve TDF change-detection accuracy compared with existing LULC methods.

## Materials and methods

### Semi-supervised algorithm design

**Fig. 1:**
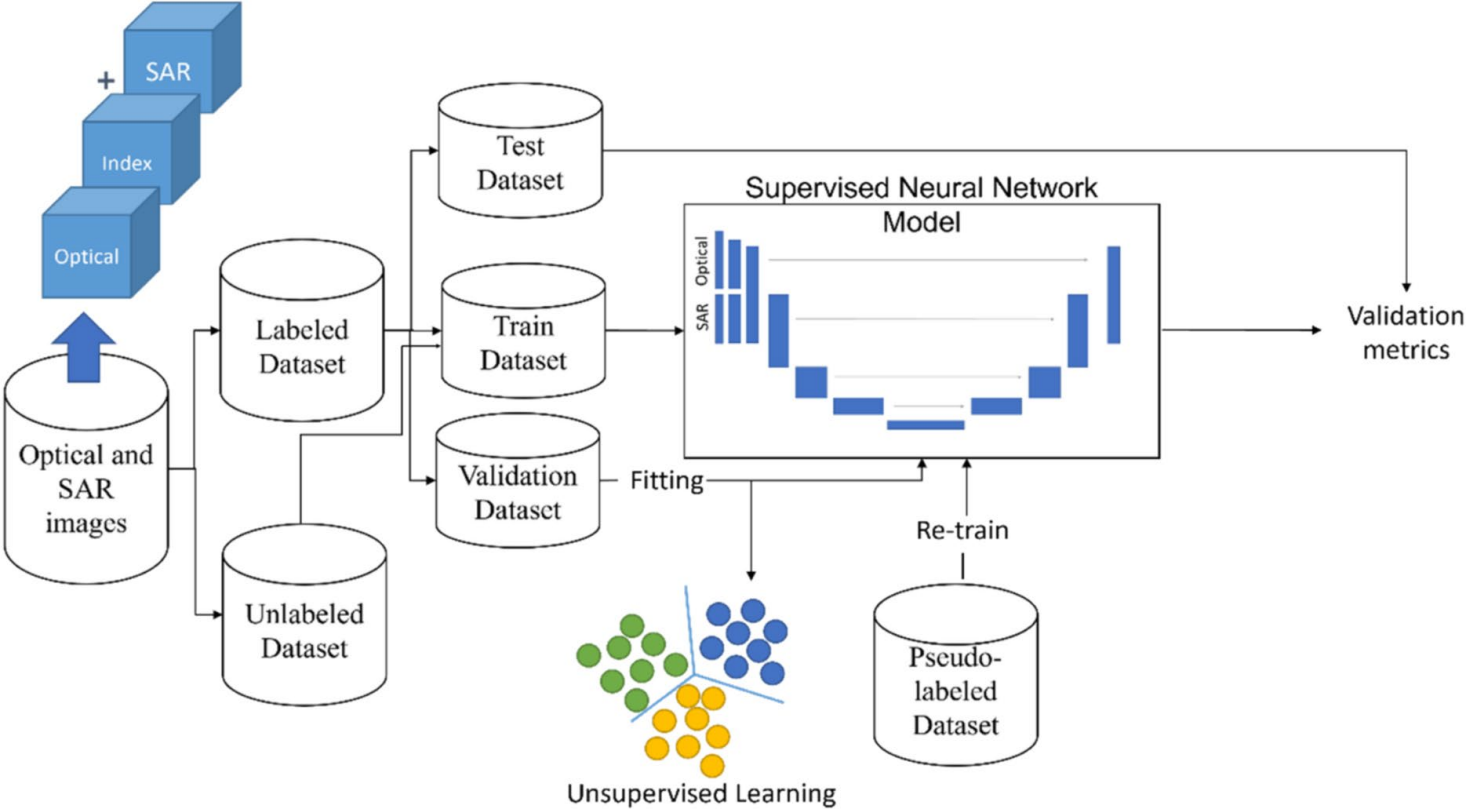
Proposed SSL framework to integrate optical and SAR images for LULC

SSL is a powerful machine learning tool that combines labeled and unlabeled data to create more robust and accurate models (Yang et al., 2022). It reduces the need for labeled data by iteratively training a classifier on both labeled and unlabeled samples (Chapelle et al., 2006). Figure 1 describes the proposed workflow for the SSL approach. The following sections explain how the supervised and unsupervised stages were designed and integrated into the proposed framework.

#### Supervised model

Supervised Convolutional Neural Networks (CNNs) utilize an encoder-decoder architecture, where an encoder extracts features and reduces image size, and a decoder reconstructs the image (Shelhamer et al., 2016). This encoder-decoder design is fundamental for successful CNNs. While established architectures like PSPNet and U-Net are effective for semantic segmentation tasks such as LULC (González-Vélez et al., 2022), they often require optical and radar images to be aligned and have the same spatial resolution.

To achieve resolution parity and leverage complementary features for LULC mapping, optical and radar images were aligned with different resolutions by down-sampling the optical image to match the 10 m SAR image u

sing max pooling. This preserves key spatial information. While reducing resolution, the U-Net architecture’s skip connections reintroduce fine details during the decoding stage, mitigating information loss from the pooling operation and allowing up-sampling back to 5 m resolution without significant detail compromise.

**Fig. 2:**
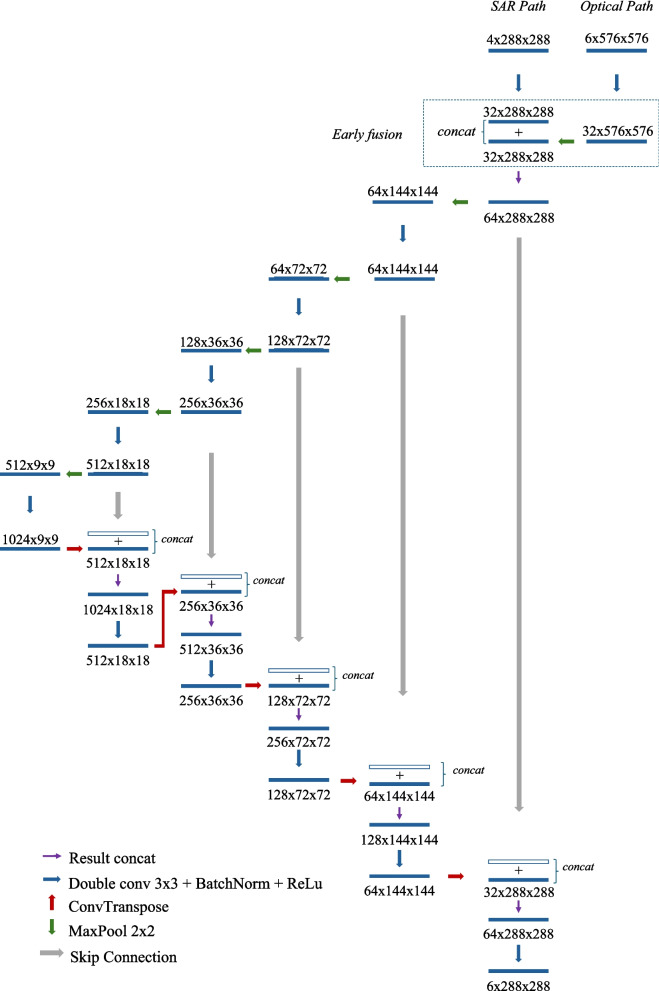
Schematic of proposed Y-Net for LULC mapping using optical and radar images, with skip connections. Fusion stage is marked in blue by dotted lines

As shown in Fig. 2, the proposed Y-Net architecture utilizes a Y-shaped encoder with distinct pathways for up-sampled optical and radar data. Features extracted from these encoders are combined early in the encoder stage (after first convolution by means of concatenation, shown inside the blue dotted box in the figure); this early feature fusion enables the network decoder to develop shared representations, leveraging the complementary aspects of SAR (structural information) and optical (spectral information) data within integrated feature maps, propagating joint features through skip connections, yielding multi-scale shared representations, thereby improving the separation of classes by combining dielectric/structural cues from SAR and spectral/phenological cues from optical improving TDF spectral differentiation to open fields, variability in forest regrowth, and seasonal leaf phenology.

Y-Net’s multi-scale structure, which includes skip connections from both encoders, further promotes a strong integration of features across different resolution levels, resulting in a more informative fused feature set for segmentation.

The proposed Y-shaped architecture offers significant flexibility. The encoder design can be modified to accommodate various input resolutions from remote sensing sensors, including commercial high-resolution imagery. This adaptability ensures that the Y-shaped architecture remains robust and scalable for different applications, from detailed urban mapping to large-scale environmental monitoring.

#### Unsupervised model

For many years, the academic community has been hugely interested in the unsupervised segmentation problem, one of the most common image processing problems. Different approaches can solve this problem. The k-means algorithm, Markov chains, graph-based methods, and normalized cuts are some of the most popular (Schmarje et al., 2021).

The unsupervised stage aims to pseudo-label satellite images for an SSL framework. Several k-means-based algorithms: traditional k-means (MacQueen, 1967), k-means++ for improved seeding and convergence (Arthur & Vassilvitskii, 2007), and PCA k-means for dimensionality reduction in high-dimensional data (Ding & He, 2004) were assessed.

Centroid-based variants were favored because k-means++ offers provably better seeding and typically faster convergence and lower distortion (Arthur & Vassilvitskii, 2007), while PCA k-means exploits the PCA–k-means equivalence to enhance separability in spectral-textural spaces (Ding & He, 2004). Moreover, these methods scale roughly linearly with the number of pixels and features, whereas spectral clustering requires eigendecomposition of large affinity matrices and becomes impractical at raster scale (von Luxburg, 2007). The number of clusters was set to *k* = 6 matching the downstream LULC class count so that pseudo-labels align with the supervised label space.

## Experiments

The experimental design is structured into seven distinct stages to progressively validate the proposed framework. Experiments 1 and 2 establish performance baselines using standard architectures (U-Net, PSPNet) on optical-only and concatenated Optical-SAR datasets. Experiment 3 evaluates the proposed Y-Net architecture’s ability to fuse multi-resolution data. Experiment 4 compares unsupervised clustering algorithms to select the optimal method for pseudo-label generation. Experiment 5 integrates these components to optimize the Semi-Supervised Learning (SSL) strategy. Finally, Experiment 6 applies the optimized model for temporal LULC change detection (2017–2021), and Experiment 7 assesses the framework’s scalability on an extended study area.

### Hardware and software

All experiments were executed on a Windows 11 Pro (64-bit) workstation comprising an Intel Core i7-10700 processor (8 cores/16 threads, 2.90 GHz base), an NVIDIA GeForce RTX 3060 graphics card with 12 GB GDDR6, 32 GB system memory, a 500 GB SSD plus a 1 TB HDD, and liquid cooling; the software stack used PyTorch 2.5.1 in a CUDA-enabled build (cuDNN 9, CUDA 12).

### Data sets

**Fig. 3:**
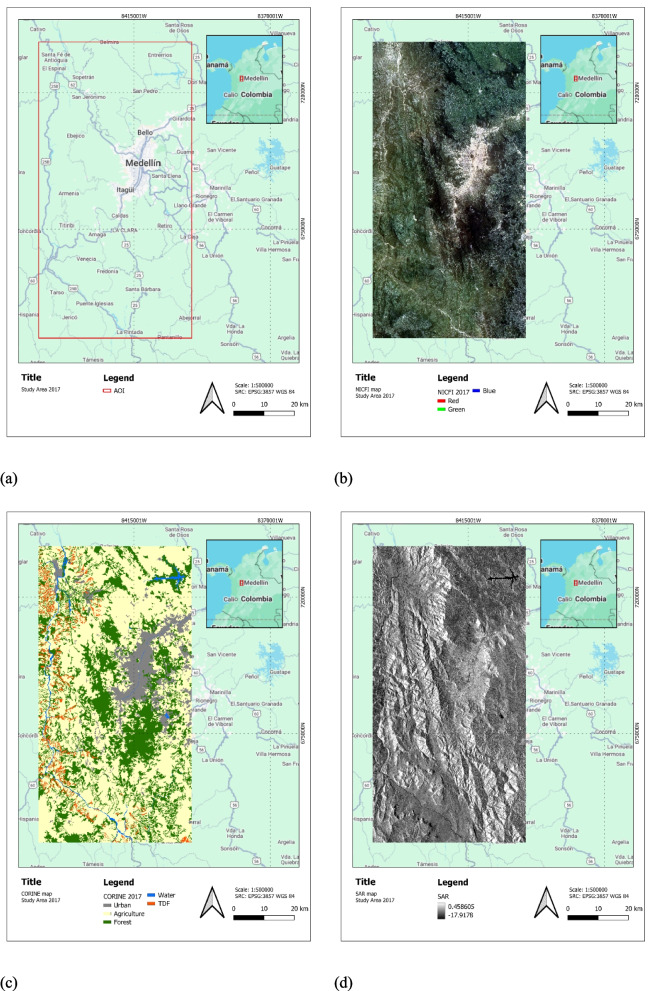
Study area: **a** localization, **b** RGB composition of the optical image from Planet Scope 2017, **c** Official IDEAM Corine Land Cover (CLC) map 2019 (derived from images of 2017) concatenated with TDF map from Instituto Alexander Von Humboldt derived from Pizano et al. (2015), and **d** SAR image 2017

The Central Andean TDF grows between 0 and 1000 m above sea level (m.a.s.l.); Antioquia is one of the regions of Colombia with the most significant TDF extension and high intervention levels. The study area (Fig. 3a) was delimited to the region between Santa Fé de Antioquia and Venecia municipalities in Antioquia’s Cauca River canyon, which contains approximately 18,000 ha of TDF. High levels of transformation have characterized this region due to pressures generated by urban development projects. This area has constant temperatures between 24 and 32 °C, an average annual precipitation of 1200 mm of rainfall, and a predominantly dry and sunny climate.

This research used several image datasets, including optical images from Sentinel-2 and the PlanetScope constellation and SAR data from Sentinel-1. Table 1 describes the images employed in detail. The images were acquired using the Google Earth Engine Online (GEE) platform.

**Table 1:** Summary of datasets used in the experiments, including sensor type, spatial resolution, spectral bands, acquisition dates, and associated experiments

Id	Sensor	Spatial resolution	Employed spectral bands	Date	Processing level/details	Experiment
1	Sentinel-2 MSI	10 m	Red – Green – Blue – NIR	2017 (6-month median composite)	L2A (Sen2Cor Surface Reflectance)	Experiments 1 and 2
2	Sentinel-1 SAR	10 m	VV – HH – VV + VH – HH + HV	2017, 2019, 2021 (Annual median composite)	GEE ARD (Terrain Corrected, Ascending Orbit)	Experiment 2, 3, 4, 5, 6, and 7
3	PlanetScope	5 m	Red – Green – Blue – NIR	2017 (6-month mosaic)	NICFI Analysis-Ready Mosaics	Experiment 3, 4, 5, 6, and 7
4	PlanetScope	5 m	Red – Green – Blue – NIR	2019 (6-month mosaic)	NICFI Analysis-Ready Mosaics	Experiments 6 and 7
5	PlanetScope	5 m	Red – Green – Blue – NIR	2021 (Annual mosaic)	NICFI Analysis-Ready Mosaics	Experiments 6 and 7

The Sentinel-2 data corresponded to analysis-ready surface reflectance mosaics for 2017 generated from atmospherically corrected images (using only the bands at 10-m spatial resolution) produced by the ESA Sen2Cor STEP Toolbox, which performs atmospheric, terrain, and cirrus correction of Level-1C data (European Space Agency, 2022). Additionally, high-resolution PlanetScope optical images are available through Norway’s International Climate and Forest Initiative (NICFI). Analysis-ready PlanetScope mosaics for 2017 (Fig. 3b), 2019, and 2021 were employed.

To produce cloud-free composite images for Sentinel-2, an automatic cloud-masking procedure was used (using GEE Sentinel-2 QA band) and mosaicked the remaining clear pixels from each six months. This modern approach ensured minimal cloud cover in each composite while preserving true surface reflectance. For the NICFI dataset 2017, 2019, and 2021, 6/12 monthly cloud-masked images were similarly combined into an annual mosaic. Each annual composite thus represents an aggregate across seasons, reducing phenological disparities between years.

It is important to note that the NICFI program provides Level 3 Analysis-Ready Mosaics rather than single scenes. The temporal resolution of these products changed over time: biannual (6-month) mosaics are available for the 2015–2020 period, while monthly mosaics are provided from September 2020 onwards. Consequently, for 2017 and 2019, the available 6-month mosaics were utilized, whereas for 2021, the available monthly mosaics were aggregated into a single annual composite. This selection was driven by the program’s data availability structure and the objective to minimize cloud cover artifacts. However, because each composite represents an aggregate across multiple months, seasonal variations are inherently averaged out. While this trade-off significantly improves cloud robustness, it can mask the fast intra-annual phenological dynamics of TDF, trading temporal fidelity for spatially consistent coverage. Finally, regarding spatial resolution, while the nominal resolution of PlanetScope (NICFI) data is ~ 4.7 m, the imagery was explicitly resampled to 5 m during the export process from GEE. This was a deliberate methodological choice to standardize the pixel size, which simplified data handling and the creation of image patches (chunks); therefore, all references to PlanetScope resolution in this study correspond to 5 m.

Sentinel-1 images (Fig. 3d) were selected for the same periods (2017, 2019, 2021). To ensure full reproducibility, all S1 data was sourced and processed using Google Earth Engine (GEE). We selected the “COPERNICUS/S1_GRD” collection, filtered for the “IW” (Interferometric Wide swath) instrument mode. Only “ASCENDING” orbits were selected to maintain geometric consistency throughout the time series. Each SAR image in the collection was already processed to an analysis-ready state (by ESA and Google), including thermal and border noise removal, radiometric calibration to backscatter, and range-doppler terrain correction using the SRTM 1 arc-second DEM, yielding geocoded images suitable for analysis.

To construct the final pseudo-quad-polarimetric composite for each year (Braun & Offermann, 2022), a temporal median reducer was applied. This process was applied separately to all available images containing VV+VH polarizations and all images containing HH+HV polarizations. The resulting four median bands (Median_VV, Median_VH, Median_HH, Median_HV) were then stacked to create a single, four-band raster (HH, HV, VV, VH) for each year. This temporal aggregation approach effectively suppresses speckle noise. Furthermore, by using the terrain-corrected GEE product and applying a temporal median reducer across all available acquisitions, geometric and radiometric variations from different incidence angles are normalized, resulting in a single, analysis-ready median composite image per year.

**Fig. 4:**
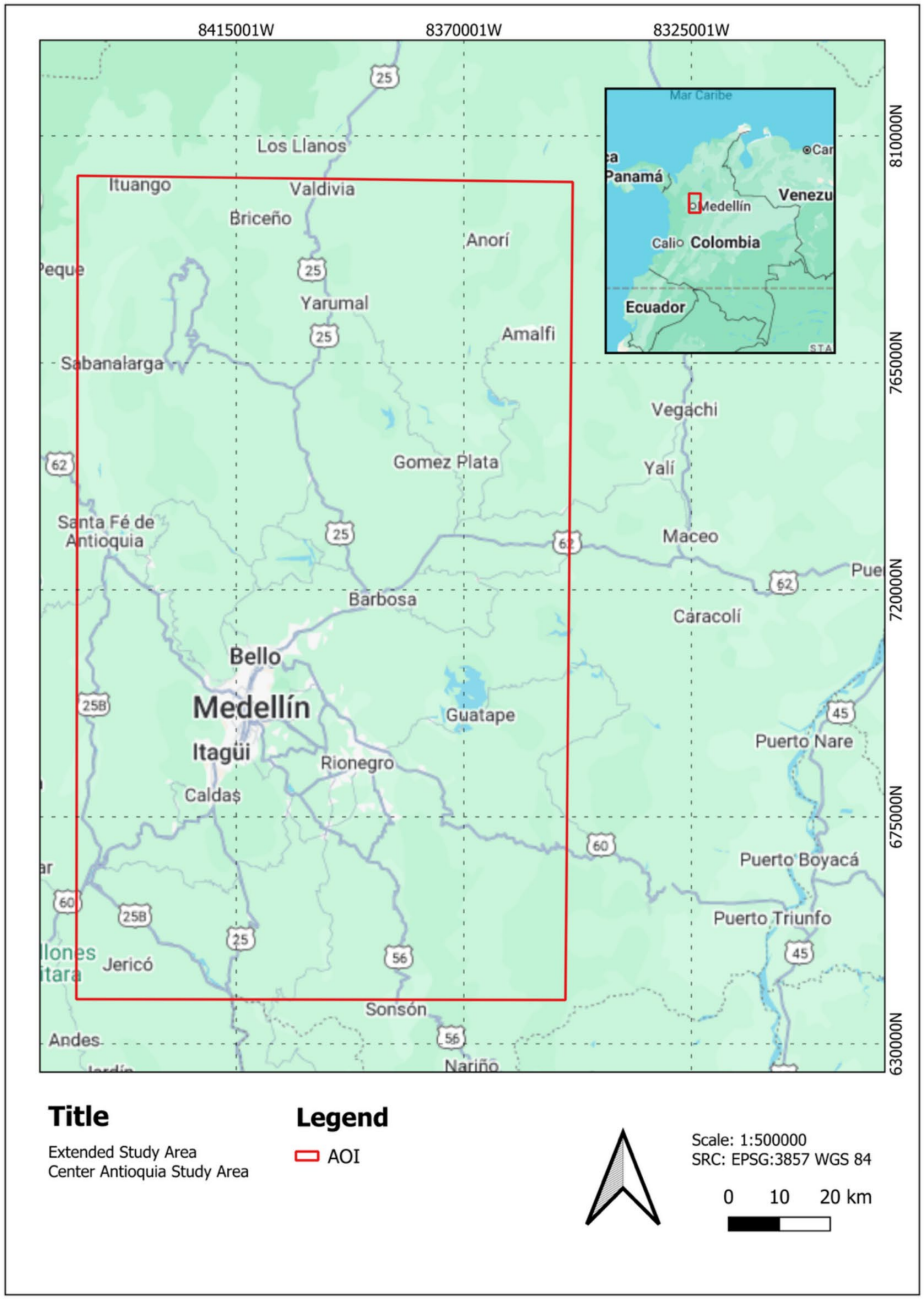
Location of the extended “Center Antioquia Study Area” (approx. 15,000 km^2^) used for the final scalability and generalization analysis (Experiment 7)

Two distinct areas were used for the experiments. For the initial model development and testing (Experiments 1–5), a Study Area corresponding to the region detailed in Fig. 3. For the final LULC change detection (Experiments 6 and 7), the full “Center Antioquia Study Area” was used. This larger region, shown in Fig. 4, covers the Cauca River Valley between the municipalities of Santa Fé de Antioquia and Venecia (approximately 15,000 km^2^). The LULC change analysis for these experiments was conducted using the PlanetScope mosaics and the Sentinel-1 SAR pseudo-quad-polarimetric median composites for 2017, 2019, and 2021, as detailed in Table 1 and the preceding data description.

A layer with the Corine Land Cover (CLC) classification map published in 2019 was acquired from the Institute of Hydrology, Meteorology and Environmental Studies (IDEAM), the latest and most up-to-date official map of LC in Colombia (scale 1:100,000; MMU 25 ha, 5 ha for artificial surfaces). The map derives from images taken in 2017 and corresponds with the classes at the first level of the adapted CLC methodology for Colombia using a combination of manually annotated images from Sentinel-1, Landsat and medium/high resolution commercial Satellites (IDEAM. 2010).

For the TDF specifically, the latest available dataset was a map published in 2020 by the Instituto Alexander Von Humboldt and the Ministry of Environment and Sustainable Development of Colombia, derived from the research conducted by Pizano et al. (2015) and represents the most updated status of TDF distribution in Colombia (Fig. 3c). Transitional or regrowth areas were labeled according to canopy structure and phenology using high‑resolution imagery. Ambiguous polygons were manually checked by experts (Pizano et al., 2015).

### Supervised experiments

In Experiment 1, to determine the best architecture for semantic segmentation in a multispectral scenario, different configurations of U-Net and PSPNet for semantic segmentation were tested in an optical-only multispectral scenario. Models using the 2017 Sentinel-2 image were trained, with each run evaluating a different encoder configuration, including ResNet-18/50/101/152 to balance capacity vs. overfitting/compute, leveraging residual learning’s depth benefits for complex TDF patterns and established transfer-learning gains on Sentinel-2, under identical U-Net/PSPNet decoders. These results established a baseline for the semantic segmentation task of TDF LULC classification.

Experiment 2 evaluated whether combining optical and radar datasets of the same resolution with a simple concatenation could enhance the baseline performance. Additionally, it assessed how increased dimensionality affected training times. Using the best-performing architecture and encoder from these experiments, a baseline for LULC classification using optical and radar imagery was established. Finally, Experiment 3 compared the proposed architecture’s (Fig. 2) performance when mixing higher-resolution optical images (5 m) with medium-resolution radar images (10 m) against the baseline architecture. For all experiments, overall accuracy and the Jaccard index, also referred to as the mean Intersection over Union (mIoU), were calculated to compare model performance.

Images were divided into chunks based on spatial resolution; images with a spatial resolution of 10 m, such as Sentinel-1 and Sentinel-2, were divided into 288 × 288 pixels, and higher-resolution images, such as NICFI at 5 m, into 576 × 576 pixels, keeping a constant ground footprint (~ 2.88 square km) across sensors, large enough to capture fragmented TDF patches and edge context, while providing sufficient receptive field for pixel-wise segmentation; maintaining GPU memory usage within bounds (NVIDIA Geforce 3060 GTX, 12 GB VRAM).

After chunking, a global normalization strategy was applied, scaling all images to [0,1]; the minimum and maximum values were calculated across all patches in the 2017 training set, and these fixed parameters were then applied to normalize the validation and test sets (including the 2019 and 2021 imagery) to ensure consistent scaling. To prepare the training, validation, and test sets, all patch identifiers were first split so that 70% formed the training and validation pool, and 30% were held out as an untouched test set. From that 70% pool, 85% of the patches were used for training and the remaining 15% for validation. This yielded 59.5% of the total data for training, 10.5% for validation, and 30% for final testing. This splitting strategy ensures the test set remains completely unseen during network training and hyperparameter tuning, while the validation set guides optimization without biasing final performance estimates. To prevent temporal leakage, the training and validation sets were constructed exclusively from 2017 imagery, whereas the 2019 and 2021 images were reserved for inference. Additionally, to minimize spatial autocorrelation, patches were grouped into non‑overlapping tiles and entire tiles were allocated to either the training or the testing set. This reduces the likelihood that adjacent pixels appear in both sets.

Multispectral indices were then calculated for each optical image, focusing on the green Normalized Difference Vegetation Index (GNDVI) and the Normalized Difference Vegetation Index (NDVI) (Muraoka et al., 2012). The selection of these two indices aimed to balance robustness and chlorophyll sensitivity: NDVI is a widely used greenness proxy, while GNDVI is more sensitive to chlorophyll and thus responsive to stress-related changes in canopy greenness, which helps with the identification of TDF, especially in the wet season. Other indices, such as the Enhanced Vegetation Index (EVI) or the Normalized Difference Water Index (NDWI), were deliberately excluded to maintain model parsimony and robustness. EVI was not considered due to concerns about its soil-adjustment factors and potential inconsistencies in cross-sensor comparability, given that the experiments involved data from both Sentinel-2 and PlanetScope. NDWI was also excluded as the primary classification challenge was discriminating TDF from other terrestrial vegetation classes (e.g., Agriculture, Forest), not from open water, for which NDVI and GNDVI were deemed more directly informative.

### Unsupervised experiments

Three unsupervised learning algorithms: k-means++, PCA k-means (Ding & He, 2004), and traditional k-means (Schmarje et al., 2021) were compared, using the validation dataset from the NICFI and SAR images (Experiment 4).

This experiment required stacking all optical, index, and SAR bands into a single 10-feature dataset for the clustering algorithms. To achieve this pixel-level alignment, and exclusively for this unsupervised experiment, the 10 m/px SAR images were up-sampled to 5 m/px using bilinear interpolation to match the optical data. While this interpolation can introduce aliasing, it was a necessary pre-processing step for the k-means methods. Additionally, exploring learned up-sampling or multiscale fusion strategies could further improve robustness and will be investigated in future work. While bilinear interpolation does not introduce new spatial information into the images, it effectively smooths the data. It increases the apparent resolution, allowing the model to learn from SAR information since it is spatially aligned with higher-resolution images.

The images were individually normalized and then stacked, building a 10-feature dataset: Red, Green, Blue, NIR, GNDVI, NDVI, VV, HH, VV + VH, and HH + HV. Four experiments were carried out for each model, randomly splitting the dataset into training (70%) and testing (30%) using the accuracy and F1 score as performance metrics.

To compute these performance metrics, the labeled validation dataset was used as ground truth. This dataset was split into the 70% training and 30% testing portions. The k-means algorithms were first trained only on the 10-feature pixel data from the 70% training set (ignoring the true labels). After the clusters were generated, a label-matching step was performed using this same 70% training set: we determined the optimal mapping between the resulting cluster indices (e.g., “Cluster 1”) and the ground-truth LULC classes (e.g., “Forest”) by assigning each cluster to the LULC class with which it had the highest overlap (a majority-vote assignment). This fixed mapping (e.g., “Cluster 1” = “Forest”) was then applied to the unseen 30% test set to calculate the final accuracy and F1 scores reported in Table 7.

### Semi-supervised experiment

The SSL approach proposed in Fig. 1 used the modified Y-Net architecture (Fig. 2) to incorporate the SAR data into the model without applying any scaling. In sharp contrast to the unsupervised experiment (Experiment 4), this architecture was specifically designed to handle multi-resolution inputs natively; thus, no scaling or interpolation was applied. The images from NICFI (5 m) and SAR (10 m) were used in this experiment (Experiment 5) in their respective separate encoder pathways, allowing the network itself to learn the feature fusion. Performance metrics such as overall accuracy and the mIoU were calculated to assess the effect of pseudo-labeling on model performance.

The Y-Net architecture was trained on datasets comprising both labeled and unlabeled images. The training set was split into subsets with varying proportions of labeled and unlabeled data, ranging from 10 to 90% unlabeled. Pseudo-labels for the unlabeled data were generated using unsupervised learning algorithms (k-means++, PCA k-means, and traditional k-means), with a majority vote determining the final pseudo-labels. These pseudo-labeled datasets were combined with the labeled data to create SSL training sets. Ranges were swept from 10 to 90% unlabeled to probe label-scarcity regimes from near-supervised to strongly SSL, keeping validation/test splits fixed across runs.

The chosen pseudo-labeling strategy trains the three variants independently and assigns each pixel the class supported by the majority, forming a simple cluster-ensemble to stabilize labels (Strehl & Ghosh, 2002). Pseudo-labels were used as supervised targets with the same loss as ground-truth labels; noise was mitigated by the 3-way majority-vote consensus retained for training. It should be noted that this was a one-shot pseudo-labeling strategy; the labels were generated once using the k-means ensemble and then remained fixed throughout the Y-Net training, rather than being iteratively updated based on the model’s predictions. Due to compute times’ concern, per-pixel confidence filtering was not applied; however, confidence-thresholded pseudo-labeling is a standard SSL practice and will be incorporated in future work (Sohn et al., 2020).

Y-Net was designed using PyTorch and trained each run for 300 epochs with a batch size of 10 images, using the AdamW optimizer with a weight decay of 5 × 10⁻^4^ and an initial learning rate of 1.58 × 10⁻^4^ that was modulated each step by a OneCycleLR scheduler. Early stopping was triggered after ten consecutive epochs without validation loss (Jaccard loss function) improvement.

### LULC detection

The LULC change detection study was performed using PlanetScope and Sentinel-1 SAR imagery from 2017, 2019, and 2021. Specifically, Experiment 6 focused on the temporal application of the optimized SSL model to generate classification maps for 2019 and 2021 within the initial Study Area to quantify TDF changes. Subsequently, Experiment 7 evaluated the model’s scalability and generalization capabilities by training on the extended ‘Center Antioquia Study Area’ (approx. 15,000 km^2^), incorporating a larger volume of unlabeled data (4,470 patches) to assess performance on a broader regional scale. To account for model stochasticity, five independent training runs were executed, each initialized with a different random seed, and the mean performance metrics are reported. Consequently, for the change detection analysis, this 5-model ensemble was utilized: each of the five trained models classified the complete image set (2017, 2019, 2021), resulting in five complete sets of classification maps.

A difference analysis was then performed using these 5 map sets to quantify changes in hectares. A pixel-wise operation was performed five times (e.g., Model 1 Map 2019 vs. Model 1 Map 2017; Model 2 Map 2019 vs. Model 2 Map 2017, etc.). The final change raster was generated by applying a majority vote to these five individual difference maps. A pixel was only considered “changed” if at least 3 of the 5 models agreed on the change. This ensemble approach ensures that the detected changes are robust and less sensitive to the inherent uncertainty of a single model’s predictions.

To analyze spatial patterns, the final majority-vote change rasters were converted into a point vector layer, enabling a precise spatial representation of individual change areas. The Getis-Ord Gi statistic (Getis & Ord, 1992), which measures the clustering and statistical significance of high (hotspots) and low (coldspots) values, was used to conduct a hotspot analysis. This analysis highlighted regions with concentrated increases or decreases in specific LC classes, revealing critical deforestation patterns, urban expansion, or agricultural development. This statistic was chosen over other spatial autocorrelation measures (e.g., Moran’s *I*) because it is specifically designed to distinguish between statistically significant spatial clusters of high values (hotspots) and low values (coldspots). This directly aligns with our goal of locating concentrated areas of TDF loss (hotspots), rather than just identifying general, non-directional clusters (i.e., high-high or low-low).

These spatial analyses are crucial for understanding the dynamics of LULC changes over time. By identifying hotspots and coldspots, conservation stakeholders and decision-makers can prioritize regions that require immediate intervention or further investigation.

## Results

### Supervised model

In Experiment 1 (optical-only classification with Sentinel-2), U-Net generally outperformed PSPNet across most metrics, achieving higher test accuracies (above 93%) and mIoU scores (above 80%). For example, U-Net with a ResNet-18 encoder achieved a test accuracy of 94.2% and a test mIoU of 82.1%, whereas PSPNet with the same encoder reached 91.7% accuracy and 75.4% mIoU. Additionally, U-Net with ResNet-18 had a relatively short training time (781.28 min), indicating an efficient balance of accuracy and speed for optical image segmentation (see Table 2).

**Table 2:** Experiment 1 results using different architectures and encoders for a supervised convolutional network for LULC classification using a dataset of optical images with multispectral indexes

Encoder type	Encoder	Train acc	Val acc	Test acc	Train mIoU	Val mIoU	Test mIoU	Time (min)
PSPNet	ResNet 18	0.939	0.907	0.917	0.821	0.786	0.754	708.23
PSPNet	ResNet 50	0.944	0.913	0.922	0.832	0.798	0.767	812.32
PSPNet	ResNet 101	0.940	0.912	0.917	0.831	0.788	0.756	815.94
PSPNet	ResNet 152	0.950	0.918	0.922	0.843	0.772	0.774	844.06
U-Net	ResNet 18	0.975	0.934	**0.942**	0.859	0.792	**0.821**	781.28
U-Net	ResNet 50	0.960	0.928	0.934	0.821	0.803	0.804	945.25
U-Net	ResNet 101	0.962	0.928	0.935	0.811	0.809	0.810	1147.04
U-Net	ResNet 152	0.957	0.925	0.930	0.801	0.796	0.801	1316.22

*acc* overall accuracy, *mIoU* mean intersection over union, *val* validation, *min* minutes. The values in bold indicate the best results

In Experiment 2 (data fusion of 10 m SAR + optical – simple concat), U-Net again outperformed PSPNet on nearly all metrics. For instance, U-Net with ResNet-18 achieved 94.2% test accuracy and 82.3% mIoU, compared to PSPNet’s 91.5% accuracy and 75.8% mIoU with the same encoder (Table 3).

**Table 3:** Experiment 2 results using different architectures and encoders for a supervised convolutional network for LULC classification with a dataset of SAR and optical concatenation

Architecture	Encoder	Train Acc	Val Acc	Test acc	Train mIoU	Val mIoU	Test mIoU	Time (min)
PSPNet	ResNet 18	0.946	0.918	0.915	0.826	0.780	0.758	1185.66
PSPNet	ResNet 50	0.949	0.919	0.921	0.821	0.781	0.769	1240.22
PSPNet	ResNet 101	0.938	0.916	0.926	0.822	0.785	0.759	1289.50
PSPNet	ResNet 152	0.944	0.914	0.927	0.843	0.797	0.776	1354.77
U-Net	ResNet 18	0.972	0.935	**0.942**	0.864	0.828	**0.823**	1234.51
U-Net	ResNet 50	0.953	0.921	0.936	0.805	0.819	0.804	1472.40
U-Net	ResNet 101	0.961	0.922	0.939	0.808	0.812	0.816	1770.25
U-Net	ResNet 152	0.944	0.933	0.938	0.827	0.823	0.803	1845.42

*acc* overall accuracy, *mIoU* mean intersection over union, *val* validation, *min* minutes. The values in bold indicate the best results

Experiment 3 compared the baseline U-Net to the proposed Y-Net (dual-stream) using 5 m optical and 10 m SAR data. Over four random trials, Y-Net consistently outperformed U-Net in all key metrics. Y-Net achieved a mean test accuracy of 96.3% and a mean mIoU of 84.7%, exceeding U-Net’s mean accuracy of 93.8% and mIoU of 82.8% (Table 4).

**Table 4:** Average performance metrics for Experiment 3 results obtained by U-Net with Resnet 18 encoder and Y-Net architectures for supervised LULC classification from SAR and optical images

Encoder type	Train Acc	Val Acc	Test acc	Train mIoU	Val mIoU	Test mIoU	Time (min)
U-Net	0.974	0.927	0.938	0.856	0.820	0.828	1183
Y-Net	0.994	0.948	**0.963**	0.879	0.838	**0.847**	1257
Difference	0.020	0.021	0.025	0.023	0.018	0.019	74

*acc* overall accuracy, *mIoU* mean intersection over union, *val* validation, *min* minutes. The values in bold indicate the best results

The confusion matrices for Experiment 3 (Tables 5 and 6) provide further insight. U-Net’s best run correctly classified approximately 94–98% of pixels in each major class (Urban, Agriculture, Forest, Water, and TDF). However, Y-Net achieved around 98% accuracy for all classes, outperforming U-Net in every category. Crucially, the per-class metrics show Y-Net achieved a high IoU of 77.8% and an F1-Score of 87.5% for the TDF class, confirming its strong performance in precisely delineating the target ecosystem. In other words, Y-Net reduced the misclassification of TDF as other classes and vice versa, evident from the higher true positive rates along the diagonal of the confusion matrix.

**Table 5:** Confusion matrix for the best result of Experiment 3 using U-Net with ResNet 18 encoder for the supervised LULC classification with SAR and optical images. (Each row is the ground truth class, and each column is the predicted class; values are percentages)

	Urban	Agriculture	Forest	Water	TDF	IoU	F1 Score
Urban	96.8%	0.7%	0.6%	0.9%	1.0%	0.867	0.928
Agriculture	1.5%	94.3%	2.9%	0.3%	1.1%	0.930	0.964
Forest	0.4%	2.4%	96.7%	0.3%	0.3%	0.907	0.951
Water	1.7%	0.3%	0.2%	97.6%	0.2%	0.733	0.846
TDF	0.2%	0.3%	1.3%	0.1%	98.2%	0.746	0.854
Overall accuracy:					0.953		
mIoU:					0.845

**Table 6:** Confusion matrix for the best result of Experiment 3 using Y-Net for the supervised LULC classification with SAR and optical images

	Urban	Agriculture	Forest	Water	TDF	IoU	F1 Score
Urban	97.9%	0.9%	0.5%	0.2%	0.5%	0.956	0.977
Agriculture	0.2%	98.0%	0.4%	0.6%	0.8%	0.975	0.987
Forest	0.3%	0.6%	98.3%	0.2%	0.7%	0.971	0.985
Water	0.6%	0.1%	0.1%	98.8%	0.4%	0.701	0.824
TDF	0.1%	0.4%	0.8%	0.1%	98.7%	0.778	0.875
Overall Accuracy:					0.981		
mIoU:					0.877		

## Unsupervised model

Table 7 compares the performance of the three unsupervised clustering algorithms. Overall, k-means++ achieved the highest mean clustering accuracy (95.5% on training data and 86.8% on testing), PCA k-means attained the highest test F1-score (79.7%), and standard k-means had the lowest performance (mean test accuracy 79.2% and F1 77.3%).

**Table 7:** Performance of unsupervised algorithms in training and testing for LULC classification (Experiment 4) was evaluated using accuracy and F1-scores

Algorithm	Train acc	Train F1-score	Test acc	Test F1-score
k-means + +	0.955	0.802	**0.868**	0.757
k-means	0.891	0.802	0.792	0.773
PCA k-means	0.886	0.824	0.823	**0.797**

*acc *overall accuracy. The values in bold indicate the best results

## Semi-supervised model

In the SSL learning experiment (Experiment 5), the Y-Net model augmented with pseudo-labeled data achieved a peak test accuracy of 97.5% and a test mIoU of 85.1% when 10% of the training data were unlabeled (with pseudo-labels). This SSL Y-Net outperformed the baseline fully supervised U-Net (93.8% accuracy and 82.8% mIoU) and even the fully supervised Y-Net (96.3% accuracy, 84.6% mIoU). As the proportion of unlabeled data increased to 20%, performance decreased slightly (to 94.7% accuracy and 84.8% mIoU), but the SSL Y-Net remained superior to the baseline models in all cases. These results, summarized in Table 8, show the impact of incorporating a moderate amount of unlabeled data (with reliable pseudo-labels) in the model’s performance.

**Table 8:** Experiment 5: Comparison among U-Net, Y-Net, and SSL Y-Net models on the optical and SAR dataset, evaluated across varying proportions of labeled and unlabeled data

% of unlabeled data	% of labeled data	Model	Train acc	Val acc	Test acc	Train mIoU	Val mIoU	Test mIoU	Time (min)
0%	100%	Baseline (U-Net)	0.974	0.927	0.938	0.856	0.820	0.828	1183
0%	100%	Y-Net	0.994	0.948	**0.963**	0.880	0.839	**0.846**	1257
10%	90%	Y-Net + SSL	0.992	0.958	0.975	0.893	0.846	0.851	1151
20%	80%	Y-Net + SSL	0.985	0.932	0.947	0.878	0.833	0.848	988
30%	70%	Y-Net + SSL	0.983	0.925	0.923	0.862	0.829	0.837	901
40%	60%	Y-Net + SSL	0.973	0.916	0.904	0.814	0.783	0.779	778
50%	50%	Y-Net + SSL	0.943	0.888	0.877	0.797	0.767	0.772	703
60%	40%	Y-Net + SSL	0.934	0.871	0.850	0.773	0.759	0.764	696
70%	30%	Y-Net + SSL	0.822	0.757	0.765	0.665	0.676	0.657	659
80%	20%	Y-Net + SSL	0.715	0.667	0.689	0.585	0.581	0.585	713
90%	10%	Y-Net + SSL	0.636	0.580	0.620	0.521	0.517	0.526	741

*acc* overall accuracy, *mIoU* mean intersection over union, *val* validation, *min* minutes

## LULC detection results

**Fig. 5:**
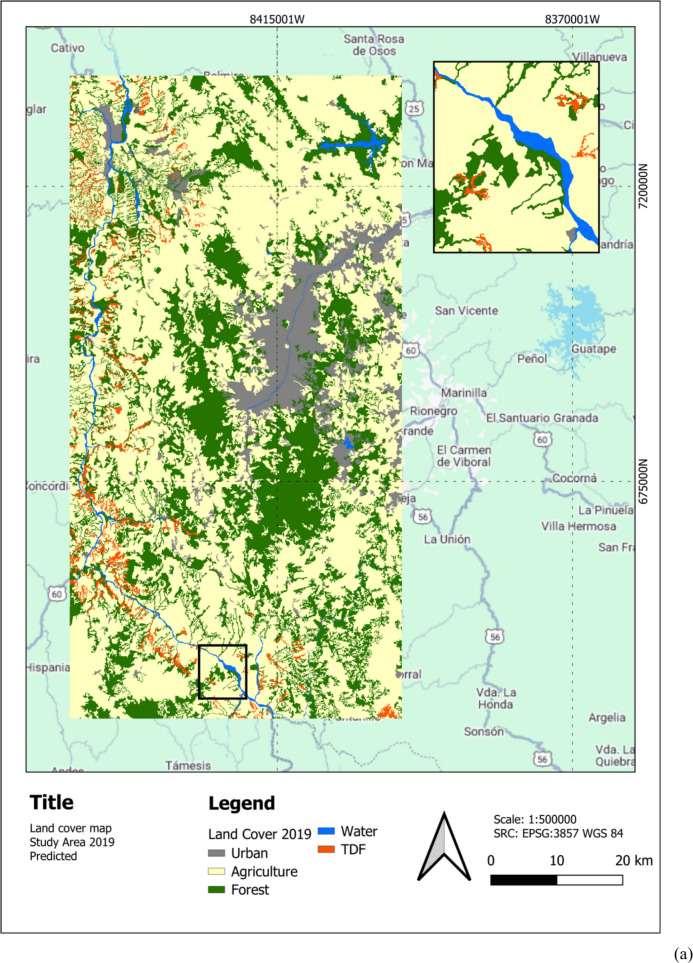

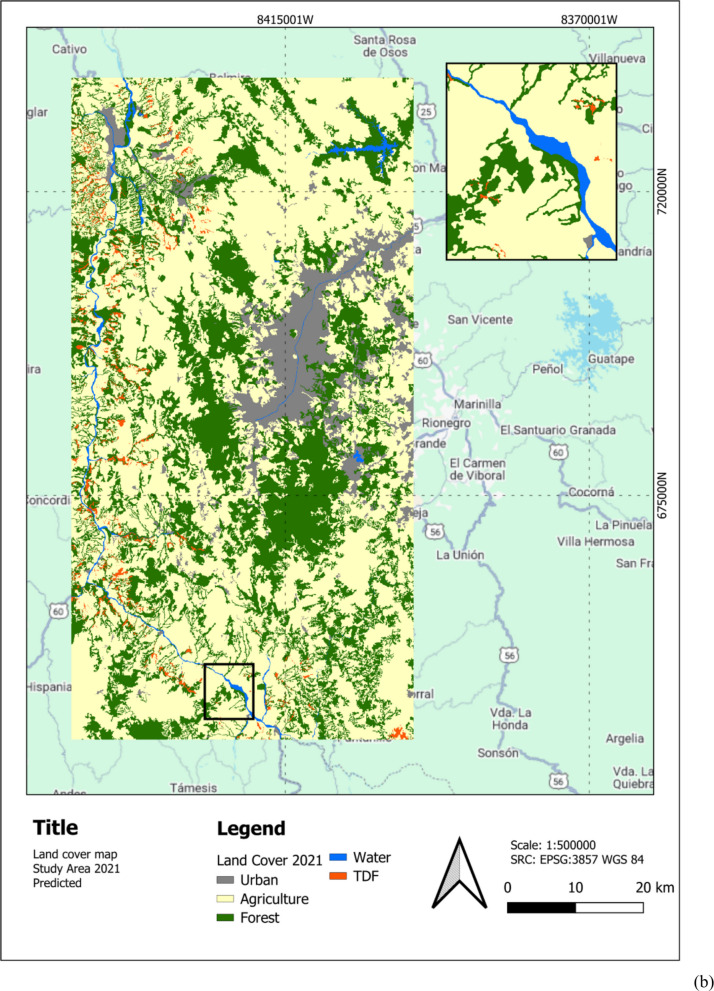
**a** Predicted LULC classification map for 2019. **b** Predicted LULC classification map for 2021. The maps use the WGS 84 projection and show urban (gray), agriculture (yellow), forest (green), water (blue), and TDF (red) classes

Figure 5 illustrates the LULC classification map for 2019 and 2021 (Experiment 6), generated with the Y-Net semisupervised methodology. The map depicts key LULC classes, including water bodies (blue), agriculture (yellow), forests (green), and TDF (red), distributed across the study area. This visualization provides a detailed spatial representation of the landscape, highlighting regions of significant TDF presence and areas of agricultural and forest land use (LU). Zoom-in shows TDF loss specially in the south between 2019 and 2021.

Using the larger Center Antioquia Study Area for change analysis, 4,470 additional unlabeled image patches alongside 561 labeled patches from the baseline experiments were incorporated to retrain the model (Experiment 7). The Experiment 7 results (extended region) indicate that the SSL Y-Net achieved a test accuracy of 95.3% and a mIoU of 88.1%. This performance is only slightly lower in accuracy than the fully supervised Y-Net’s 96.3%. These outcomes, summarized in Table 9, suggest that leveraging a large set of unlabeled data from the broader area helped capture more variability in TDF appearance, thus improving the model’s generalization.

**Table 9:** Optical + SAR dataset testing results

# of Unlabeled Optical images	# of labeled Optical images	Model	Train Acc	Val Acc	Test acc	Train mIoU	Val mIoU	Test mIoU	Time (min)
0	561	Baseline (U-Net)	0.974	0.927	0.938	0.856	0.820	0.828	1183
0	561	Y-Net	0.994	0.948	0.963	0.879	0.838	0.847	1257
4470	561	Y-Net + semi-supervised	0.994	0.964	0.953	0.906	0.883	0.881	1865

*acc* overall accuracy, *mIoU* mean intersection over union, *val* validation, *min* minutes

Table 10 presents the confusion matrix for the Y-Net + SSL architecture with the inclusion of 4,470 additional unlabeled images. It showcases the model’s performance across multiple LU and LULC classes, including urban, agriculture, forest, water, and TDF. The diagonal entries represent the percentage and absolute count of correctly classified pixels for each class, while off-diagonal entries indicate misclassifications. The per-class metrics confirm the model’s robustness, achieving an F1-Score of 87.9% and an IoU of 93.6% for the TDF class, even in this larger, more complex study area, increasing the performance reported in Experiment 3 for both U-Net and Y-Net.

**Table 10:** Confusion matrix for experiment 7 results using semi-supervised Y-Net with SAR and optical images

	Urban	Agriculture	Forest	Water	TDF	IoU	F1 Score
Urban	96.7%	1.2%	0.6%	0.5%	1.0%	0.835	0.910
Agriculture	2.1%	94.5%	3.1%	0.1%	0.3%	0.928	0.963
Forest	0.5%	3.2%	96.0%	0.2%	0.2%	0.898	0.946
Water	0.6%	0.3%	0.2%	98.8%	0.1%	0.853	0.921
TDF	0.1%	0.2%	0.8%	0.1%	98.8%	0.879	0.936
Overall Accuracy:					0.953		
mIoU:					0.888		

From the classification maps obtained from the SSL Y-Net, changes in hectares of TDF cover between 2017, 2019, and 2021 (Table 11) were identified by difference pixel-wise. The final map reveals a significant decline in TDF cover over the years, with a 7.8% reduction observed from 2017 to 2019 and an additional 5.4% loss between 2019 and 2021. By 2021, the TDF area was estimated at 18,586 hectares, compared to 21,307 hectares in 2017.

**Table 11:** Y-Net + Semisupervised predictions over images from 2019 and 2021 for TDF

Year	Hectares of TDF	TDF LULC change	Source	Error margin (ha) derived from TDF % error from Y-Net (Table 11)
2017	21,307	NA	Pizano et al. (2015)	NA
2019	19,645	−7.8%	Y-Net + semisupervised	± 235 (1.2%)
2021	18,586	−5.4%	Y-Net + semisupervised	± 223 (1.2%)

**Fig. 6:**
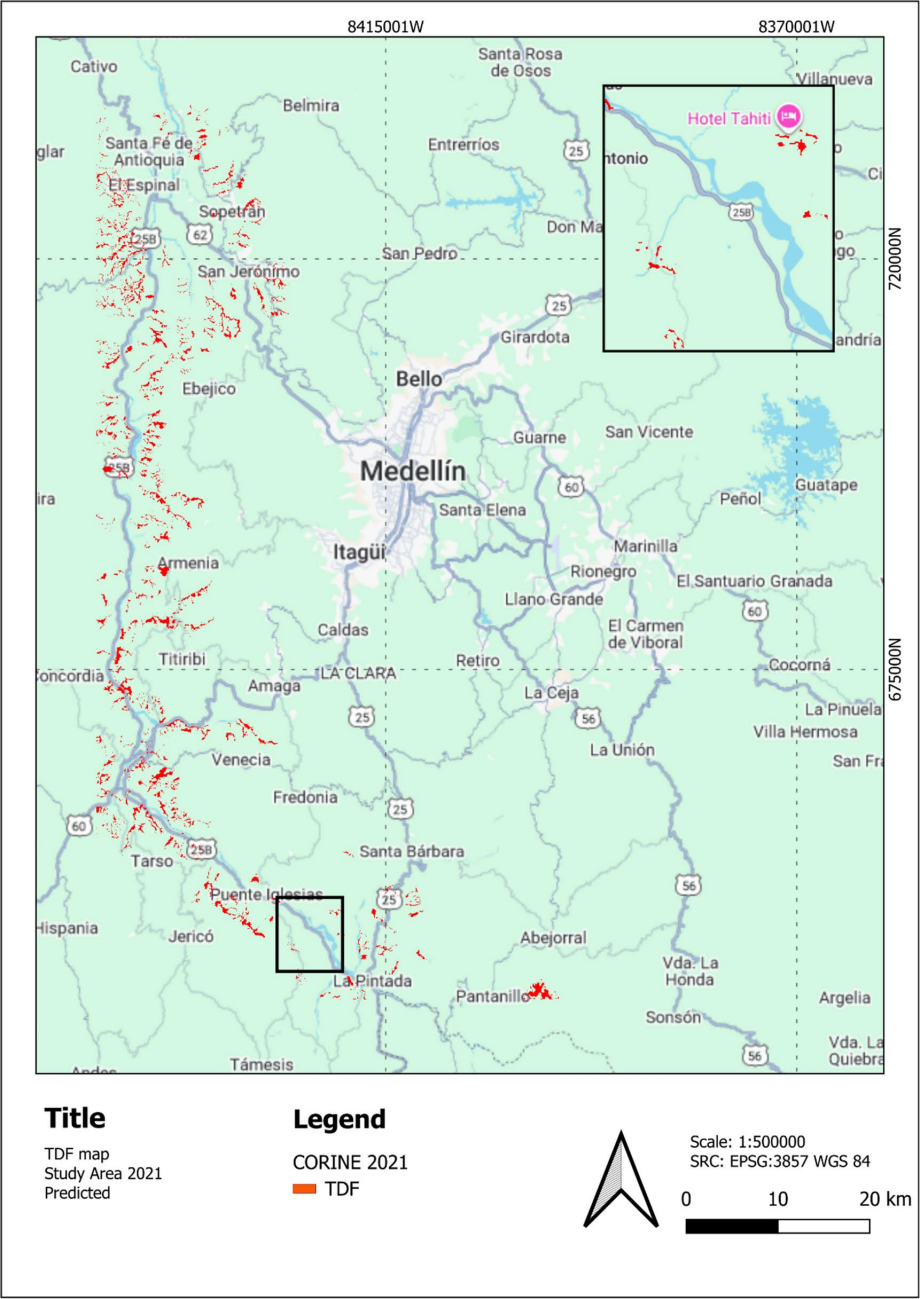
Study area with predicted TDF for 2021 in the Cauca River Valley region in Antioquia, Colombia

**Fig. 7:**
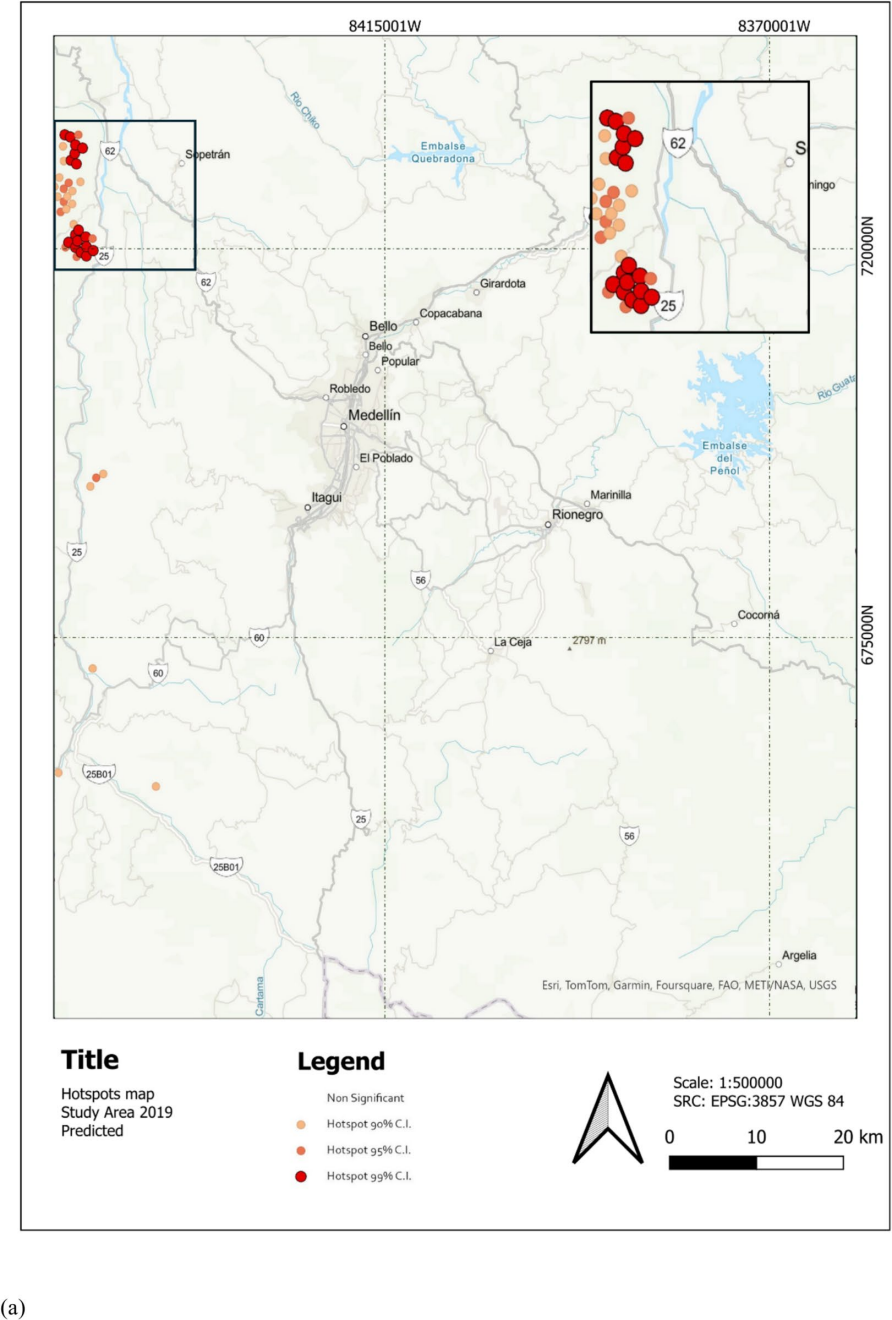

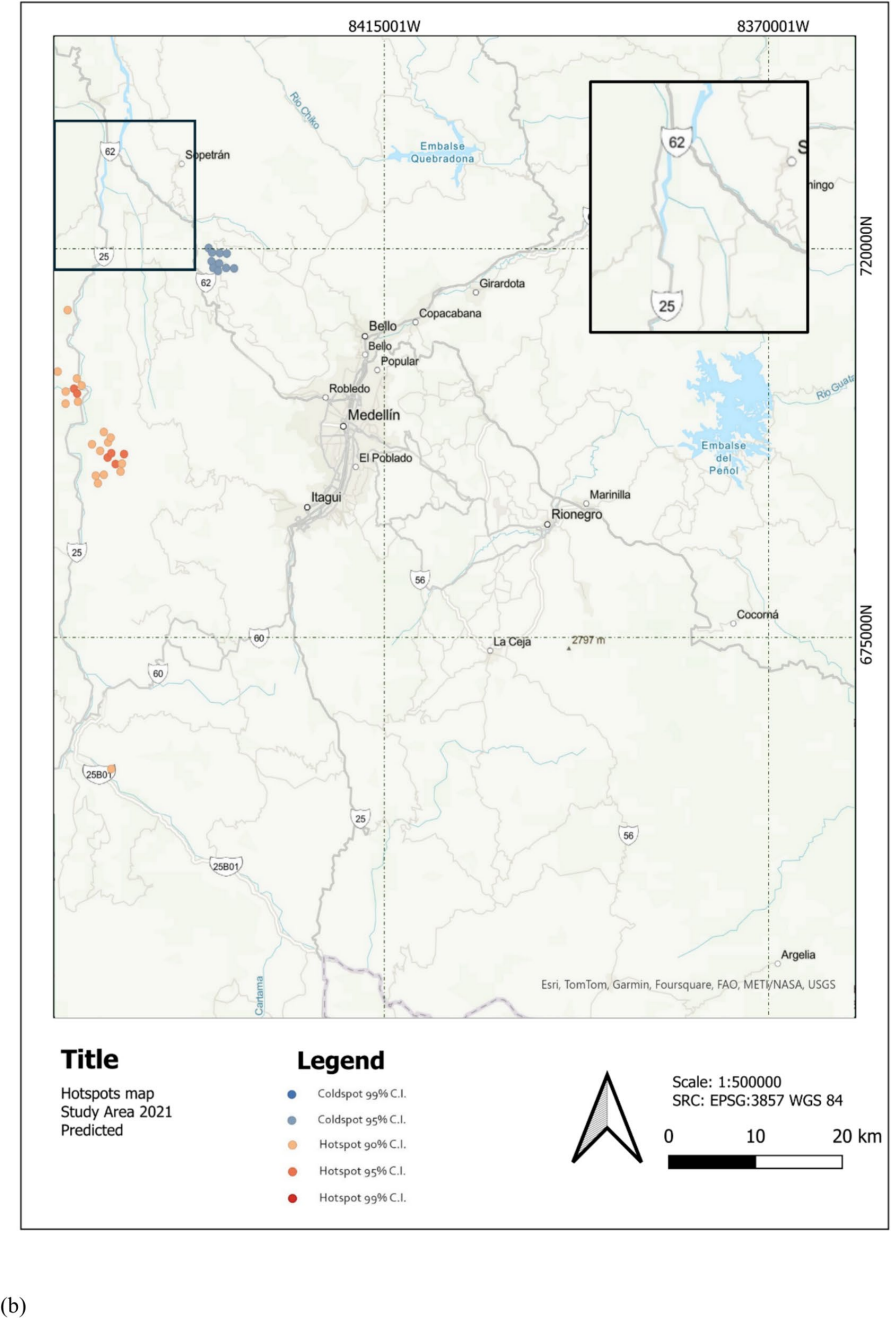
Hotspot analysis of TDF LC changes between **a** 2017–2019 and **b** 2019–2021. Areas in red indicate significant hotspots of TDF loss, highlighting regions with concentrated deforestation activity

Figure 6 shows the study area with an overlay of the predicted TDF LC for 2021. The map highlights the spatial distribution of TDF (in red) within the region, providing a visual representation of its extent and fragmentation. Zoom-in shows the same area as Fig. 6 showcasing TDF loss, especially in the south between 2019 and 2021. Moreover, Fig. 7 shows hotspot analyses of TDF LC changes over two distinct periods: (a) 2017–2019 and (b) 2019–2021. The maps identify significant hotspots of TDF loss, represented in red, highlighting regions experiencing concentrated deforestation activity.

## Discussion

### Benchmarking architectures and modalities

Experiments 1 and 2 (Tables 2 and 3) showed that U-Net, with ResNet-18 as the encoder, was the best LULC classification architecture for optical and SAR + optical images. This configuration outperformed all other tested architectures. One advantage of U-Net with ResNet-18 is its relatively low training time, which makes it suitable for large-scale applications. This architecture took 781.28 min to train in Experiment 1, showing ResNet-18’s efficiency, consistent with Rakhlin et al. (2018) lightweight U-Net accuracy in LC classification, was significantly surpassed by the Y-Net model, which exceeded their reported 85.5% accuracy and 0.664 IoU, further validating the benefits of employing ResNet-18 for LULC classification demonstrating that U-Net’s performance advantage persisted even when combining SAR and optical imagery.

Experiment 2 (Table 3) highlights the value of combining SAR and optical imagery for LULC classification. Among the evaluated architectures, PSPNet (ResNet-18) had the fastest training time (1185.66 min), highlighting computational demands. U-Net (ResNet-18) was the best performer, accurately combining data sources, positioning U-Net with lightweight encoders as a practical choice for applications needing computational efficiency and performance. These findings support previous research stressing the benefits of fusing SAR and optical data for classification. Solórzano et al. (2021) showed that U-Net architectures incorporating Sentinel-1 and Sentinel-2 imagery enhanced classification accuracy, achieving an overall accuracy of 76% and improved F1-scores for classes like old-growth plantations. Similarly, Sun and Zheng (2022) used advanced CNN architectures like HRNet and PSPNet on multiband data, achieving high segmentation accuracy while illustrating the computational trade-offs of complex networks.

Experiment 2 also reveals several significant observations that build on previous patterns shown in Experiment 1. Firstly, even though the PSPNet architectures employing deeper ResNet encoders, 50, 101, and 152, show slight improvements in test accuracies or mIoU values compared to the shallower ResNet 18 encoder, the gains are modest.

Similar trends are observable in the U-Net variants, where deeper encoders can achieve comparable or slightly better performance but significantly heighten the training complexity and resource demands. While increasing model complexity may offer minor performance enhancements, these are often disproportionate to the substantial rise in computational requirements (Yuan et al., 2022). This observation aligns with Pashaei et al. (2020) findings, who demonstrated that U-Net architectures with lightweight encoders, such as ResNet-34, achieved high accuracy in LC classification tasks while maintaining reasonable training times. Their study emphasizes that increasing model depth does not always lead to significant performance gains and can introduce substantial computational overhead.

### Evaluation of the Y-Net and semi-supervised framework

Experiment 3 (Table 4) demonstrated that the Y-Net architecture consistently outperforms U-Net in semantic segmentation tasks involving SAR and optical images, achieving a test accuracy of 0.963 and a test mIoU of 0.847. When compared to similar studies, Y-Net achieved competitive performance. Wittstruck et al. (2024) employed a 3D U-Net with multi-stage feature fusion to integrate SAR and multispectral images for crop-type mapping, achieving a test accuracy of 0.9454 and a test mIoU of 0.794. While Wittstruck et al.'s approach highlights the effectiveness of multi-stage fusion for crop classification, Y-Net's multi-scale fusion strategy achieves higher accuracy and segmentation quality, showcasing its robustness across broader LULC categories indicating that the dual-encoder fusion of high-resolution optical and medium-resolution radar data (without resampling) improved classification performance.

Furthermore, Y-Net’s test accuracy of 0.963 improves the results from both Wittstruck et al. (2024) and Hafner et al. (2021), reinforcing the advantages of its ability to fuse data from differing resolutions and extract complementary features at multiple scales.

As shown in Table 6, the proposed model accurately identifies key LC classes, achieving a classification accuracy of 98.7% for TDF. This is particularly noteworthy given TDF’s spectral and structural similarities to other vegetation types, which often complicate differentiation through conventional approaches. Additionally, the model performs exceptionally well in other categories, achieving accuracies of 98.8% for Water and 97.9% for Urban areas. In comparison, Wagner et al. (2023) employed a U-Net model to map forest and deforestation trends in Mato Grosso, Brazil, using PlanetScope images. While their approach achieved a high F1 score of over 0.98, it focused on a binary Forest/Non-Forest classification.

Regarding Unsupervised methods presented in Table 7, PCA k-means attained the highest test F1-score (79.7%), indicating it balanced precision and recall slightly better for some classes. In contrast, standard k-means had the lowest performance (mean test accuracy 79.2% and F1 77.3%), suggesting it struggled to segregate the complex feature space as effectively. Therefore, k-means++ was deemed the most reliable for generating pseudo-labels in this context and was used for subsequent SSL labeling. However, a majority vote algorithm was implemented between the pseudo labels generated by each k-means algorithm to select the pseudo label for the SSL algorithm. This strategy leverages the diversity and complementarity of different k-means models to minimize error propagation.

Table 8 illustrates the results of experiments that assessed the baseline method, supervised U-Net, and Y-Net, with the proposed SSL approach. The results indicate that the best SSL method performs best with a 30–70% unlabeled to labeled data ratio (test accuracy of 0.923 and mIoU of 0.837). This reflects a slight decrease of 0.040 (4.15%) in accuracy and 0.009 (1.06%) in mIoU compared to the fully supervised Y-Net. The results demonstrate the efficiency of combining Y-Net with SSL learning. A significant advantage of this approach lies in its ability to reduce training times. For instance, training the Y-Net model with 70% unlabeled data required 659 min, a reduction from the 1257 min needed for the fully supervised Y-Net model. These findings align with the results of Pareek et al. (2024), where incorporating SSL significantly reduced computational costs while maintaining comparable performance levels. This approach reduces the dependency on manual labeling while maintaining competitive accuracy, making it particularly valuable for remote sensing tasks in data-scarce regions. While the experiments compared different architectures and training strategies, a full component‑wise ablation was beyond the scope of this study and remains as future work.

The results for LULC detection (Table 9) demonstrated the Y-Net’s efficiency and scalability when paired with the SSL method. Utilizing an unlabeled dataset including 4470 optical images with the fully labeled training set extended the training duration but improved overall performance (time increased to 1865 min compared to 1257 min of the supervised Y-Net). This increase in time represents a reasonable compromise considering the significant increase in training data without incurring extra labeling expenses. The model achieved a test accuracy of 95.3% and a test mIoU of 0.881, demonstrating the model’s ability to leverage additional pseudo-labeled data to improve feature learning and generalization. This allows CNNs to be trained with high volumes of unlabeled images to improve generalization. These results align with Ma et al. (2023), who also demonstrated the effectiveness of SSL in LULC classification. Ma et al.’s confidence-guided approach achieved an accuracy of 90.3% and an F1-score of 88.7% on the ISPRS Potsdam dataset.

### LULC dynamics and TDF conservation

Figure 6 illustrates the LULC final classification results produced by the Y-Net architecture with the SSL approach compared to the corresponding ground truth. While some inaccuracies in the ground truth labels may exist due to inherent dataset limitations, the model demonstrates remarkable predictive capability. The two TDF maps were compared to identify the changes between the two dates in Table 11. Five inferences were made yearly, determining TDF loss pixels by majority vote. The resulting images show that the TDF decreased between 2017 and 2019 by 7.8%, and between 2019 and 2021 by 5.4%, suggesting that the loss of cover has slowed after 2020, which contrasts with the findings of (Céspedes et al., 2022). Table 11 also reports a 1.2% confidence interval for the predicted TDF by Y-Net from Table 10 areas in 2019 and 2021.

Figure 7a displays the results of a hotspot analysis of LULC change for TDF between 2017 and 2019. The most prominent hotspots, shown in red, are concentrated in the northwest region of the study area, while smaller and less intense clusters are scattered in other areas. This concentration of hotspots indicates regions experiencing notable land transformations, such as deforestation, urbanization, or land-use conversion.

The temporal component is key here, as it reflects the intensity of changes over a relatively short period (2017–2019), signaling recent pressures that warrant immediate attention (Calderón-Caro et al., 2024). The spatial pattern of LULC changes shows an apparent clustering of hotspots near Santa Fe de Antioquia, a historic municipality in the Antioquia department. The proximity of these hotspots to key infrastructure highlights the role of improved accessibility in accelerating land-use change and urban growth in this region (Ocampo et al., 2018).

The spatial clustering observed in the map aligns with broader trends seen in areas experiencing rapid land-use change, where urban expansion and tourism-related activities tend to fragment critical habitats (Rivas & Navarro-Cerrillo, 2024).

Figure 7b shows LULC change hotspots and cold spots for TDF from 2019 to 2021. Compared to 2017–2019, the hotspot distribution has shifted, with significant clusters now between Santa Fe de Antioquia and Bolombolo, while earlier northwest concentrations have decreased. Cold spots have appeared between Medellín and San Jerónimo, an area typically with low population density and economic activity linked to transportation.

This change highlights the dynamic nature of land-use shifts due to socio-economic disruptions like the ones caused by COVID-19 (Amador-Jiménez et al., 2020); with halted tourism and construction, demand for land-use changes dropped. The economic downturn stalled infrastructure and housing development in hotspot areas, redistributing land-use pressures (Bolton, 2021).

### Limitations and future directions

While effective, the Y-Net architecture is more complex than a standard U-Net and requires careful tuning (learning rates, batch normalization between the two streams, etc.). It was found that an improper training setup could cause one encoder stream to dominate the other. Balancing contributions from optical and SAR branches is an area for further research (perhaps through learnable weights or adaptive fusion techniques).

Despite these caveats, the approach employed successfully mapped and detected changes in a challenging environment. The integration of SAR was critical, as many optical images of the study area during wet season months were unusable due to clouds. The SAR provided year-round monitoring capability, and its sensitivity to structure complemented the spectral information from optical imagery. While effective, this framework has several limitations that must be acknowledged:

- First, the pseudo-labeling strategy did not use confidence filtering due to computational costs. Moreover, the reliance on three variants of the same centroid-based algorithm (k-means) creates a risk of reinforcing systematic bias rather than correcting for it; future work should use more diverse unsupervised ensembles.
- Second, significant uncertainty stems from the reference datasets. A scale mismatch exists between our 5 m predictions and the reference maps, particularly the CLC map, which introduces boundary uncertainties. This also leads to uncertainty propagation: any errors in the 2017 baseline maps are inevitably carried forward into the change detection analysis.
- Third, the TDF reference class itself includes ecologically ambiguous transitional and regrowth areas, creating inherent label uncertainty. Finally, our use of annual seasonal compositing, while necessary for managing cloud cover, averages out intra-annual phenological dynamics, a known limitation for TDF mapping.
- Fourth, a significant limitation is the lack of an independent, external validation dataset of expert-labeled change polygons; such a dataset was not available for our study area and would be required for a “gold standard” assessment of the final change maps.
- Finally, it must be noted that per-pixel probabilistic uncertainty (e.g., from the softmax output) was not propagated into the change detection analysis, which is a limitation when reporting exact hectare changes. However, our ensemble method of using a majority vote from 5 independent model trainings was implemented as a practical alternative to account for model uncertainty (i.e., stochasticity from training), ensuring that the reported changes are robust and not an artifact of a single model run.

In comparison to other recent studies, this framework achieved high accuracy. For example, Hafner et al. (2021) reported using a dual-stream U-Net on Sentinel-1 and Sentinel-2 for urban change detection with an overall accuracy around 95%, similar to this paper’s results but in a binary urban-change context. The multi-class mapping used here, including an ecosystem like TDF at 96% accuracy, is notable. Additionally, Castro-Ospina et al. (2023) used soundscape data to identify TDF transformation, an entirely different modality, with supervised learning. While their approach is innovative for detection, it lacks the spatial explicitness of satellite imagery. A combination of acoustic monitoring and this remote sensing approach could be a promising future direction for cross-validation and increased confidence in results.

## Conclusions

The proposed Y-Net architecture, by effectively fusing SAR and Optical imagery, achieved high accuracy (95.3% overall accuracy, 88.1% mIoU) for complex LULC mapping within the Cauca River Valley, Colombia. While these high accuracies should be interpreted with caution given the limitations of the reference datasets (e.g., scale mismatch and temporal ambiguity, as noted in the Discussion), this advancement signifies progress in remote sensing applications for heterogeneous landscapes such as TDF.

This study highlights a critical 5.4% loss in TDF area between 2019 and 2021 (with an error margin derived from the model’s 1.2% TDF misclassification rate), emphasizing the urgent need for effective conservation strategies. These results confirm the importance of monitoring this endangered ecosystem as it faces threats from deforestation, agricultural expansion, and urbanization.

Furthermore, the SSL framework proved to be a robust method for combining limited labeled data with large volumes of unlabeled data. This contribution is particularly valuable not only for this study area but for TDF monitoring in general, where the scarcity of labeled, cloud-free optical images remains a critical barrier to advancing environmental monitoring.

Future research directions should focus on integrating this remote sensing framework with rigorous field-based validation, analyzing the socioeconomic drivers of the detected TDF changes, and assessing scalability to other TDF regions to better inform conservation policy.

## Data Availability

No datasets were generated or analysed during the current study.

## References

[CR1] Aide, T. M., Clark, M. L., Grau, H. R., López‐Carr, D., Levy, M. A., Redo, D., Bonilla‐Moheno, M., Riner, G., Andrade‐Núñez, M. J., & Muñiz, M. (2012). Deforestation and reforestation of Latin America and the Caribbean (2001–2010). *Biotropica,**45*(2), 262–271. 10.1111/j.1744-7429.2012.00908.x

[CR2] Amador-Jiménez, M., Millner, N., Palmer, C., Pennington, R. T., & Sileci, L. (2020). The unintended impact of Colombia’s COVID-19 lockdown on forest fires. *Environmental and Resource Economics,**76*(4), 1081–1105. 10.1007/s10640-020-00501-5 32836864 10.1007/s10640-020-00501-5 PMC7416588

[CR4] Arthur, D., & Vassilvitskii, S. (2007). k-means++: The advantages of careful seeding. In *Proceedings of the 18th Annual ACM-SIAM Symposium on Discrete Algorithms (SODA)* (pp. 1027–1035). https://theory.stanford.edu/~sergei/papers/kMeansPP-soda.pdf. Accessed June 2024.

[CR6] Bolton, L. (2021). The economic impact of COVID-19 in Colombia. 10.19088/k4d.2021.073

[CR7] Braun, A., & Offermann, E. (2022). Polarimetric information content of Sentinel-1 for land cover mapping: An experimental case study using quad-pol data synthesized from complementary repeat-pass acquisitions. *Frontiers in Remote Sensing*. 10.3389/frsen.2022.905713

[CR8] Calderón-Caro, J., Morales-Gómez, L. M., Gutiérrez-Vélez, V. H., González-Caro, S., & Benavides, A. M. (2024). Modeling proximate causes of deforestation in Antioquia, Colombia. *Regional Environmental Change*. 10.1007/s10113-024-02302-8

[CR9] Castillo, M., Rivard, B., Sánchez-Azofeifa, A., Calvo-Alvarado, J., & Dubayah, R. (2012). LIDAR remote sensing for secondary tropical dry forest identification. *Remote Sensing of Environment,**121*, 132–143. 10.1016/j.rse.2012.01.012

[CR10] Castro, K. L., Sanchez-Azofeifa, G. A., & Rivard, B. (2003). Monitoring secondary tropical forests using space-borne data: Implications for Central America. *International Journal of Remote Sensing,**24*(9), 1853–1894. 10.1080/01431160210154056

[CR11] Castro-Ospina, A. E., Rodríguez-Buritica, S., Rendon, N., Velandia-García, M. C., Isaza, C., & Martínez-Vargas, J. D. (2023). Identification of tropical dry forest transformation from soundscapes using supervised learning. *Communications in computer and information science* (pp. 173–184). 10.1007/978-3-031-32213-6_13

[CR12] Céspedes, J., Sylvester, J. M., Pérez-Marulanda, L., Paz-Garcia, P., Reymondin, L., Khodadadi, M., Tello, J. J., & Castro-Nunez, A. (2022). Has global deforestation accelerated due to the COVID-19 pandemic? *Journal of Forestry Research,**34*(4), 1153–1165. 10.1007/s11676-022-01561-7 10.1007/s11676-022-01561-7 PMC9666988 36405883

[CR13] Chapelle, O., Schlkopf, B., & Zien, A. (2006). Semi-supervised learning. *In the MIT Press eBooks*. 10.7551/mitpress/9780262033589.001.0001

[CR14] Chen, B., Wang, L., Fan, X., Bo, W., Yang, X., & Tjahjadi, T. (2023). Semi-FCMNet: Semi-supervised learning for forest cover mapping from satellite imagery via ensemble self-training and perturbation. *Remote Sensing,**15*(16), 4012. 10.3390/rs15164012

[CR15] Clerici, N., Calderón, Ca. V., & Posada, J. M. (2017). Fusion of Sentinel-1A and Sentinel-2A data for land cover mapping: A case study in the lower Magdalena region, Colombia. *Journal of Maps,**13*(2), 718–726. 10.1080/17445647.2017.1372316

[CR16] De La Barreda-Bautista, B., A, A., Couturier, S., & Luis, J. (2011). Tropical dry forests in the global picture: The challenge of remote sensing-based change detection in tropical dry environments. In InTech eBooks. 10.5772/24283

[CR17] Ding, C., & He, X. (2004). K-means clustering via principal component analysis. Proc. 21st Int. Conf. Mach. Learn, 29. 10.1145/1015330.1015408

[CR18] Gaur, S., & Singh, R. (2023). A comprehensive review on land use/land cover (LULC) change modeling for urban development: Current status and future prospects. *Sustainability,**15*(2), 903. 10.3390/su15020903

[CR19] Getis, A., & Ord, J. K. (1992). The analysis of spatial association by use of distance statistics. *Geographical Analysis,**24*(3), 189–206. 10.1111/j.1538-4632.1992.tb00261.x

[CR20] González-Vélez, J. C., Martinez-Vargas, J. D., & Torres-Madronero, M. C. (2022). Land cover classification using CNN and semantic segmentation: A case of study in Antioquia, Colombia. *Communications in computer and information science* (pp. 306–317). 10.1007/978-3-030-99170-8_22

[CR21] Hafner, S., Nascetti, A., Azizpour, H., & Ban, Y. (2021). Sentinel-1 and Sentinel-2 data fusion for urban change detection using a dual stream U-Net. *IEEE Geoscience and Remote Sensing Letters,**19*, 1–5. 10.1109/lgrs.2021.3119856

[CR22] Hafner, S., Nascetti, A., Azizpour, H., & Ban, Y. (2023). Semi-supervised urban change detection using multi-modal Sentinel-1 SAR and Sentinel-2 MSI data. *Remote Sensing,**15*(21), Article 5135. 10.3390/rs15215135

[CR23] Helmer, E. H. (2000). The landscape ecology of tropical secondary forest in Montane Costa Rica. *Ecosystems,**3*(1), 98–114. 10.1007/s100210000013

[CR24] Hernández-Stefanoni, J. L., Castillo-Santiago, M. Á., Mas, J. F., Wheeler, C. E., Andres-Mauricio, J., Tun-Dzul, F., George-Chacón, S. P., Reyes-Palomeque, G., Castellanos-Basto, B., Vaca, R., & Dupuy, J. M. (2020). Improving aboveground biomass maps of tropical dry forests by integrating LiDAR, ALOS PALSAR, climate and field data. *Carbon Balance and Management*. 10.1186/s13021-020-00151-6 10.1186/s13021-020-00151-6 PMC7392681 32729000

[CR25] Herrera-Ruiz, V., Perez-Guerra, J., Martínez-Vargas, J. D., Gonzalez-Velez, J. C., & Torres-Madronero, M. C. (2023). Fusion of optical and radar data by aggregation into a single feature space for LULC classification. In Communications in computer and information science (pp. 25–34). 10.1007/978-3-031-47372-2_3

[CR26] IDEAM (Institute of Hydrology, Meteorology and Environmental Studies). (2010). *National Land Cover Legend: CORINE Land Cover methodology adapted to Colombia (1: 100,000 scale)*. IDEAM.

[CR27] Kechagias-Stamatis, O., & Aouf, N. (2021). Automatic target recognition on synthetic aperture radar imagery: A survey. *IEEE Aerospace and Electronic Systems Magazine,**36*(3), 56–81. 10.1109/maes.2021.3049857

[CR28] Lan, H., Jiang, D., Yang, C., Gao, F., & Gao, F. (2020). Y-Net: Hybrid deep learning image reconstruction for photoacoustic tomography in vivo. *Photoacoustics,**20*, Article 100197. 10.1016/j.pacs.2020.100197 32612929 10.1016/j.pacs.2020.100197 PMC7322183

[CR30] Li, L., Mu, X., Jiang, H., Chianucci, F., Hu, R., Song, W., Qi, J., Liu, S., Zhou, J., Chen, L., Huang, H., & Yan, G. (2023). Review of ground and aerial methods for vegetation cover fraction (fCover) and related quantities estimation: Definitions, advances, challenges, and future perspectives. *ISPRS Journal of Photogrammetry and Remote Sensing,**199*, 133–156. 10.1016/j.isprsjprs.2023.03.020

[CR31] Ma, W., Oktay, K., & Paul L. R., (2023). *Confidence-guided semi-supervised learning in land cover classification.* arXiv.Org. May 17, 2023. https://arxiv.org/abs/2305.10344. Accessed Nov 2024.

[CR32] MacQueen, J. (1967). Some methods for classification and analysis of multivariate observations. In Proceedings of the Fifth Berkeley Symposium on Mathematical Statistics and Probability, Volume 1 (pp. 281–297). University of California Press.

[CR33] Miles, L., Newton, A. C., DeFries, R. S., Ravilious, C., May, I., Blyth, S., Kapos, V., & Gordon, J. E. (2006). A global overview of the conservation status of tropical dry forests. *Journal of Biogeography,**33*(3), 491–505. 10.1111/j.1365-2699.2005.01424.x

[CR34] Mohsenifar, A., Mohammadzadeh, A., Moghimi, A., & Salehi, B. (2021). A novel unsupervised forest change detection method based on the integration of a multiresolution singular value decomposition fusion and an edge-aware Markov Random Field algorithm. *International Journal of Remote Sensing,**42*(24), 9376–9404. 10.1080/01431161.2021.1995075

[CR35] Mullissa, A., Reiche, J., & Herold, M. (2023). Deep learning and automatic reference label harvesting for Sentinel-1 SAR-based rapid tropical dry forest disturbance mapping. *Remote Sensing of Environment,**298*, Article 113799. 10.1016/j.rse.2023.113799

[CR36] Muraoka, H., Noda, H. M., Nagai, S., Motohka, T., Saitoh, T. M., Nasahara, K. N., & Saigusa, N. (2012). Spectral vegetation indices as the indicator of canopy photosynthetic productivity in a deciduous broadleaf forest. *Journal of Plant Ecology,**6*(5), 393–407. 10.1093/jpe/rts037

[CR37] Murphy, P. G., & Lugo, A. E. (1986). Ecology of tropical dry forest. *Annual Review of Ecology and Systematics,**17*(1), 67–88. 10.1146/annurev.es.17.110186.000435

[CR38] Ocampo, L. M. E., Arenas, C. M., Zuluaga, E. P., & Escobar, L. F. G. (2018). La transformación del paisaje de Santa Fe de Antioquia: reconfiguración del centro histórico y su entorno natural. Perspectiva Geográfica, 23(1). 10.19053/01233769.7087

[CR39] Pareek, P., Kaarthik, S., Deepjyoti, D., & Sidhant, M. (2024). *Optimization proxies using limited labeled data and training time -- A semi-supervised Bayesian neural network approach*. arXiv.Org. October 4, 2024. https://arxiv.org/abs/2410.03085. Accessed June 2025.

[CR40] Pashaei, M., Kamangir, H., Starek, M. J., & Tissot, P. (2020). Review and evaluation of deep learning architectures for efficient land cover mapping with UAS hyper-spatial imagery: A case study over a wetland. *Remote Sensing,**12*(6), Article 959. 10.3390/rs12060959

[CR41] Pizano, C., González-M, R., Castaño Naranjo, A., Cuadros, H., Jurado-B, R., López Camacho, R., Perez, K., Rodríguez, N., Rojas, A., Toro, J., & García, H. (2015). *A national assessment of the successional stage of and anthropogenic pressures on tropical dry forests in Colombia*. Accessed Nov 2024.

[CR42] Rakhlin, A., Davydow, A., & Nikolenko, S. (2018). Land cover classification from satellite imagery with U-Net and Lovász-Softmax Loss. *IEEE/CVF Conference on Computer Vision and Pattern Recognition Workshops (CVPRW),**2022*, 257–2574. 10.1109/cvprw.2018.00048

[CR43] Rivas, C. A., & Navarro-Cerrillo, R. M. (2024). Forest fragmentation and connectivity in South American dry forests. *Biodiversity and Conservation,**33*(11), 3015–3037. 10.1007/s10531-024-02894-x

[CR44] Sánchez-Cuervo, A. M., Aide, T. M., Clark, M. L., & Etter, A. (2012). Land cover change in Colombia: Surprising forest recovery trends between 2001 and 2010. *PLoS ONE,**7*(8), Article e43943. 10.1371/journal.pone.0043943 22952816 10.1371/journal.pone.0043943 PMC3430633

[CR46] Schmarje, L., Santarossa, M., Schroder, S., & Koch, R. (2021). A survey on semi-, self- and unsupervised learning for image classification. *IEEE Access,**9*, 82146–82168. 10.1109/access.2021.3084358

[CR47] Shelhamer, E., Long, J., & Darrell, T. (2016). Fully convolutional networks for semantic segmentation. *IEEE Transactions on Pattern Analysis and Machine Intelligence,**39*(4), 640–651. 10.1109/tpami.2016.2572683 27244717 10.1109/TPAMI.2016.2572683

[CR48] Shimizu, K., Ota, T., & Mizoue, N. (2019). Detecting forest changes using dense Landsat 8 and Sentinel-1 time series data in tropical seasonal forests. *Remote Sensing,**11*(16), 1899. 10.3390/rs11161899

[CR49] Sohn, K., Berthelot, D., Li, C.-L., Zhang, Z., Carlini, N., Cubuk, E. D., Kurakin, A., Zhang, H., & Raffel, C. (2020). FixMatch: Simplifying semi-supervised learning with consistency and confidence. *Advances in Neural Information Processing Systems (NeurIPS),**33*, 596–608.

[CR50] Solórzano, J. V., Mas, J. F., Gao, Y., & Gallardo-Cruz, J. A. (2021). Land use land cover classification with U-Net: Advantages of combining Sentinel-1 and Sentinel-2 imagery. *Remote Sensing,**13*(18), 3600. 10.3390/rs13183600

[CR51] Strehl, A., & Ghosh, J. (2002). Cluster ensembles—A knowledge reuse framework for combining multiple partitions. *Journal of Machine Learning Research,**3*, 583–617.

[CR52] Sun, Y., & Zheng, W. (2022). HRNet- and PSPNet-based multiband semantic segmentation of remote sensing images. *Neural Computing and Applications*. 10.1007/s00521-022-07737-w

[CR53] von Luxburg, U. (2007). A tutorial on spectral clustering. *Statistics and Computing,**17*(4), 395–416. 10.1007/s11222-007-9033-z

[CR54] Wagner, F. H., Dalagnol, R., Silva-Junior, C. H. L., Carter, G., Ritz, A. L., Hirye, M. C. M., Ometto, J. P. H. B., & Saatchi, S. (2023). Mapping tropical forest cover and deforestation with planet NICFI satellite images and deep learning in Mato Grosso State (Brazil) from 2015 to 2021. *Remote Sensing,**15*(2), 521. 10.3390/rs15020521

[CR55] Wittstruck, L., Jarmer, T., & Waske, B. (2024). Multi-stage feature fusion of multispectral and SAR satellite images for seasonal crop-type mapping at regional scale using an adapted 3D U-Net model. *Remote Sensing,**16*(17), 3115. 10.3390/rs16173115

[CR56] Xie, Y., Sha, Z., & Yu, M. (2008). Remote sensing imagery in vegetation mapping: A review. *Journal of Plant Ecology,**1*(1), 9–23. 10.1093/jpe/rtm005

[CR57] Yang, X., Song, Z., King, I., & Xu, Z. (2022). A survey on deep semi-supervised learning. *IEEE Transactions on Knowledge and Data Engineering,**35*(9), 8934–8954. 10.1109/tkde.2022.3220219

[CR58] Yuan, H., Zhang, Z., Rong, X., Feng, D., Zhang, S., & Yang, S. (2023). MPFFNet: Lulc classification model for high-resolution remote sensing images with multi-path feature fusion. *International Journal of Remote Sensing,**44*(19), 6089–6116. 10.1080/01431161.2023.2261153

[CR59] Yuan, W., Wang, J., & Xu, W. (2022). Shift pooling PSPNet: Rethinking PSPNet for building extraction in remote sensing images from entire local feature pooling. *Remote Sensing,**14*(19), 4889. 10.3390/rs14194889

